# IL-4 alpha chain receptor (IL-4Rα) polymorphisms in allergic bronchopulmonary sspergillosis

**DOI:** 10.1186/1476-7961-4-3

**Published:** 2006-02-17

**Authors:** Alan P Knutsen, Barbara Kariuki, Judy D Consolino, Manoj R Warrier

**Affiliations:** 1 Division of Allergy/Immunology, Saint Louis University Health Sciences Center and St. Louis, Missouri, USA; 2 Division of Pulomology, Saint Louis University Health Sciences Center, St. Louis, Missouri, USA; 3 Professor Pediatrics, Director Allergy & Immunology, Pediatric Research Institute, 3662 Park Avenue, St. Louis, MO 63110, USA

## Abstract

**Background:**

Allergic bronchopulmonary aspergillosis occurs in 7–10% of cystic fibrosis (CF) and 1–2% of asthmatic patients. HLA-DR restriction and increased sensitivity to IL-4 stimulation have been proposed as risk factors in these populations.

**Objective:**

We examined for the presence of IL-4 receptor alpha chain (IL-4Rα) single nucleotide polymorphisms (SNPs) in ABPA and whether these accounted for increased sensitivity to IL-4 stimulation.

**Methods:**

One extracellular (ile75val) and four cytoplasmic IL-4Rα SNPs were analyzed in 40 CF and 22 asthmatic patients and in 56 non-ABPA CF and asthmatic patients. Sensitivity to IL-4 stimulation was measured by induction of CD23 expression on B cells.

**Results:**

IL-4Rα SNPs were observed in 95% of ABPA patients. The predominant IL-4Rα SNP was the extracellular IL-4Rα SNP, ile75val, observed in 80% of ABPA patients.

**Conclusion:**

The presence of IL-4Rα SNPs, principally ile75val, appears to be a genetic risk for the development of ABPA.

## Background

Allergic bronchopulmonary aspergillosis (ABPA) is a Th2 cell mediated hypersensitivity lung disease due to bronchial colonization with *A. fumigatus *that affects approximately 1–2% of asthmatic and 7–9% of cystic fibrosis (CF) patients [1,2]. The immunologic hallmarks of ABPA are elevated IgE antibodies to *A. fumigatus*, elevated total serum IgE levels, and peripheral blood and bronchial eosinophilia. A sequelae of ABPA is bronchiectasis and/or pulmonary fibrosis which progressively compromises respiratory function increasing morbidity and mortality. Sensitization to *A. fumigatus *is common in asthmatic and CF patients, yet only a small percentage develop ABPA. We hypothesize that ABPA patients have genetic risk factors which predispose for the development of ABPA. Chauhan et al [3-5] found that ABPA patients have increased frequency of HLA-DR2 and DR5. However, only a minority of atopic patients with these genotypes develop ABPA.

In earlier studies, we reported that ABPA patients demonstrated increased sensitivity to IL-4 stimulation as measured by up-regulation of CD23 on their B-cells [6,7]. Previous studies have reported that single nucleotide polymorphisms (SNP) of the IL-4 alpha receptor (IL-4Rα) is associated with up-regulation of IL-4 stimulation [8-16].

In this study to explain this observation of increased sensitivity to IL-4 stimulation, we examined IL-4 receptor alpha chain (IL-4Rα) single nucleotide polymorphisms (SNP). The frequency of IL-4Rα SNPs was present in approximately 95% of ABPA patients, and the predominant SNP was ile75val in the IL-4 binding region. We propose that increased sensitivity to IL-4 in conjunction with HLA-DR2/DR5 restriction to *Aspergillus *antigens in ABPA patients result in increased B-cell activity, monocyte/dendritic cell phenotype that polarizes *Aspergillus*-specific Th2 responses.

## Methods

### Patients

The study population consisted of 40 CF and asthmatic ABPA patients and 56 non-ABPA CF and asthmatic control patients. The study was approved by the Saint Louis University Institutional Review Board. Criteria for ABPA in asthmatic patients are those described previously by Greenberger [1]. Recently, a Cystic Fibrosis Foundation Consensus Conference proposed diagnostic criteria for the diagnosis of ABPA in CF patients that was used for this study [2]:

1. Clinical deterioration (cough, wheeze, exercise intolerance, exercise induced asthma, deterioration of pulmonary function, increased sputum).

2. Serum IgE level ≥500 IU/ml.

3. IgE anti-*Aspergillus *antibody. Either immediate cutaneous reactivity to *Aspergillus *or presence of IgE anti-Af antibodies by ELISA.

4. IgG anti-*Aspergillus *antibody. Either precipitating antibodies to *Aspergillus *or presence of IgG anti-Af antibodies by ELISA.

5. Abnormal chest radiograph and/or high-resolution computerized tomography (infiltrates, bronchiectasis, plugging, or change from previous radiograph), as read by the radiologist.

### IL-4Rα genotyping by PCR with sequence-specific primers and direct sequence analysis

Genomic variants in IL-4Rα gene was identified by direct sequencing using PCR-based method for single nucleotide polymorphism (SNP). Genomic variants of IL-4Rα were numbered on the basis of their location in IL-4Rα mRNA sequence of gene bank accession number X52425. Five previously reported IL-4Rα variants ile75val, glu400ala, cys431arg, ser503pro and gln576arg (numbering including the 25 amino acid signal peptide) were genotyped. Presence of polymorphisms and its allelic variants were determined by direct PCR-SSP protocols with slight modifications [17].

Genotyping for the ile75val SNP of the IL-4Rα gene was determined by PCR amplification. A 755 bp long region of IL-4Rα was amplified using the following primers: forward 5' ATGCAGAGCTTATGTTGTTCTA3' and reverse 5' TTCCTCCTGCTGTTGCTATGA3'. PCR reactions for identification of IL-4Rα variants glu400ala, cys431arg, ser503leu and gln576arg were conducted by amplifying a 991 bp long fragment using the following primers: forward 5' CTGTTTTCTGGAGCACAACAT 3' and reverse 5' CTTGAGAAGGCCTTGTAACCA 3'. Allele specific primers were used to amplify specific regions of IL-4 receptors containing the alleles of interest using a thermal cycler (Perkin Elmer Model 9700, Cetus, Norwalk, CT) and polymerase (AmpliTaq Gold, Perkin Elmer) as previously described by Bux et al [18]. Thermal cycling conditions for first PCR amplification of genomic fragments was as follows: Each DNA sample was subjected to 40 cycles of denaturation at 95°C for 45 s, annealed at 60°C for 45 s and extension at 72°C for 90 s. PCR products were visualized on 1% agarose gel with ethidium bromide. A second PCR was performed for direct sequencing of amplified fragments using the Big Dye Terminator Kit (Perkin-Elmer Applied Biosystems, Foster City, CA) and subsequent analysis on a capillary automated sequencer CEQ 2000XL (Beckman Coulter, Fullerton, CA). The presence of IL-4Rα nucleotide polymorphisms were examined using the NCBI Blast program (; accession number 33833); homozygous/heterozygous SNPs were detected on the nucleotide chromatograph.

### IL-4 induction of B-cell CD23 expression

Peripheral blood mononuclear cells (PBMC) were isolated from venous blood by Ficoll-Hypaque density centrifugation as previously described [6,7]. PBMC were cultured at 1 × 10^6 ^cells/ml in 1 ml of RPMI 1640 supplemented with 10% FCS for 48 hours at 37°C in a 6% CO_2 _humidified atmosphere. The cultures were stimulated with rhuIL-4 (PeproTech, Inc) at 1, 5, 10 and 25 ng/ml. After 48 hours, the cells were washed and analyzed by flow cytometry.

### Flow cytometry

PBMC prior to culture and after culture were analyzed for induction of cell surface CD23 expression on B-cells, and T-cells analyzed for induction of cytoplasmic cytokine expression as previously described [7]. For cultures stimulated with IL-4 and IL-13, PBMC were washed and stained with murine monoclonal antibody to CD23-PE and CD20 Per-CP (Becton Dickinson). PBMCs were washed and fixed with 1% PBS buffered paraformaldehyde. Forward and side-scatter was performed to gate on the live lymphocyte population and further gated on CD20^+ ^cells for analysis using the CellQuest MacIntosh program (Becton Dickinson). A minimum of 10,000 cells were counted. Quantibrite PE flow cytometry beads (Becton Dickinson) were used to quantitate the number of CD23 receptors per B-cell for each experiment. The beads contain a given number of PE molecules per bead. A linear equation was calculated from which the number of CD23 receptors per cell was extrapolated, and the total number of CD23 receptors expressed per B-cell expressed.

### Statistics

Analysis of the frequency of IL-4Rα SNPs was analyzed by chi square analysis and used to compare ABPA versus the non-ABPA patients. CD23 expression was analyzed by Student's t-test with unequal variance. Based on the published data, we estimate that there will need to be 30 patients in each group to have a 95% power to detect a mean difference of 9.48%, statistically significant with P value (alpha) = .05 (two-tailed), using GraphPad StatMate software package.

## Results

### Patients

The demographic characteristics of the patients are presented in Table 1. There were 14 CF and 26 asthmatic ABPA patients; there were 33 CF and 23 asthmatic non-ABPA patients. The sex distribution was equal in the asthmatic and CF ABPA patients compared to the non-ABPA patients, 65% versus 64%. The demongraphic of the asthmatic and CF ABPA patients was comparable except that asthmatic ABPA patients were older than CF ABPA patients, 41 versus 12 years old, and this also observed for the non-ABPA patients. IgE reactivity to *A. fumigatus *was identified in 100% of ABPA patients and in 41% of non-ABPA patients; IgG reactivity was determined by ELISA assay and was identified in 100% of ABPA and 88% of non-ABPA patients. Serum IgE levels were markedly elevated in ABPA patients compared to non-ABPA patients, 2617 ± 2275 versus 87 ± 133 IU/ml (P < 0.01).

**Table 1:** Demographics of asthmatic and cystic fibrosis patients with allergic bronchopulmonary aspergillosis (ABPA).

**Study**	**ABPA (40)**	**Non-ABPA (56)**
CF	14 (35%)	33 (59%)
Asthma	26 (65%)	23 (41%)
Age, yrs	24 ± 18	27 ± 12
Sex, % male	65%	64%
IgE, IU/ml	2617 ± 2275	87 ± 133
Reactivity to *A. fumigatus*		
Positive Af skin test	40 (100%)	23 (41%)
IgG anti-Af antibody	40 (100%)	49 (88%)

Abbreviations: ABPA, allergic bronchopulmonary aspergillosis; CF, cystic fibrosis; Af, *Aspergillus fumigatus*.

Data presented as number of patients and in parentheses percentage. For age and IgE, data presented as Mean ± SD.

### IL-4Ra SNPs

The frequency of IL-4 Rα SNPs was significantly increased in ABPA patients compared to non-ABPA patients, 95% versus 61% (p = 0.0001) (Table 2). The predominant IL-4Rα SNP observed was ile75val in IL-4 binding region. This was observed in 80% of ABPA patients compared to 54% of non-ABPA patients (p = 0.008). The frequencies of IL-4Rα SNPs were comparable comparing asthmatic and CF ABPA patients: IL-4Rα SNPs 93% and 96% and ile75val 79% and 81%, respectively. Furthermore, the ile75val SNP was homozygous in 43% of ABPA patients compared to 11% of non-ABPA patients (p = 0.0003). An additional extracellular SNP, asn98thr, was identified in a CF ABPA patient as an isolated polymorphism. We previously reported this patient, and this was associated with increased sensitivity to IL-4 stimulation [19]. One or more cytoplasmic IL-4Rα SNPs were observed in 38% of ABPA patients compared to 27% of non-ABPA patients (p = ns). The combination of ile75val and cytoplasmic SNPs were not significantly increased in ABPA patients compared to non-ABPA patients, present in 28% and 21%, respectively.

**Table 2:** IL-4Rα single nucleotide polymorphisms in asthmatic and cystic fibrosis patients with allergic bronchopulmonary aspergillosis (ABPA).

**Study**	**Number of Patients**		P*
	**ABPA (40)**	**Non-ABPA (56)**	
Extracellular IL-4Rα SNPs			
ile75val	32 (80%)	30 (54%)	0.008
asn98thr	2 (5%)	4 (8%)	ns
Homozygous ile75val	17 (43%)	6 (11%)	0.0003
			
Cytoplasmic IL-4Rα SNPs			
glu400ala	5 (13%)	3 (5%)	ns
cys431arg	3 (8%)	4 (7%)	ns
ser503pro	9 (23%)	10 (18%)	ns
gln576arg	8 (20%)	14 (25%)	ns
			
Any IL-4Rα SNP	38 (95%)	34 (61%)	0.0001
Extracellular and Cytoplasmic IL-4Rα SNP	11 (28%)	12 (21%)	ns

Abbreviations: IL-4Rα, interleukin 4 receptor alpha chain; SNP, single nucleotide polymorphism; ABPA, allergic bronchopulmonary aspergillosis; ns, not significant.

In parentheses, percentage (%) of patients.

* Chi square analysis comparing ABPA versus Non-ABPA patients.

### Up-regulation of CD23 by IL-4 stimulation

**Figure 1:**
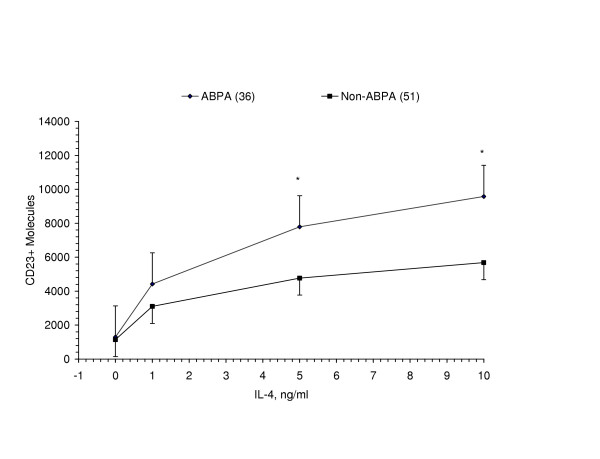
*IL-4 induction *of *CD23+ molecules on CD20+B-cells*. Following IL-4 stimulation, ABPA patients had a significantly increased rate of CD23+ expression per B-cell compared to non-ABPA patients at 5 ng/ml and 10 ng/ml (p < 0.02*, < 0.02*). Data presented as Mean ± SE.

The up-regulation of CD23 molecules on B-cells by IL-4 stimulation is shown in Figure 1. In the absence of IL-4, the number of CD23 molecules decreased after 48 hours in media and was comparable in both groups. With IL-4 stimulation, the slope of increased number of CD23 molecules per B-cell was significantly greater in ABPA patients compared to non-ABPA patients (p < 0.01). Furthermore, the number of CD23 molecules per B-cell was increased in ABPA patients at IL-4 concentrations of 1 ng/ml, 5 ng/ml and 10 ng/ml. Up-regulation of CD23 expression was observed for each of the IL-4Rα SNPs.

## Discussion

A key question in the development of ABPA in CF and asthmatic patients is why do only 1–2% and 7–9% of this at risk population develop ABPA. Chauhan et al [3-5] observed that patients with asthma and CF who expressed HLA-DR2 and/or DR5 (and perhaps HLA-DR4 or DR7) but lacked HLA-DQ2 were at increased risk for ABPA after exposure to *A. fumigatus*. Within HLA-DR2 and HLA-DR5, restricted genotypes, in particular, HLA-DR2 B1*1501 and 1503 and HLA-DR5 B1*1101 and B1*1103, provide high relative risk. On the other hand, 40% to 44% of non-ABPA atopic *Aspergillus*-sensitive individuals have the HLA-DR2 and/or DR5 type. These results demonstrating HLA-DR restriction are similar to those found with purified house dust mite allergens [20-22]. Thus, certain genotypes of HLA-DR2 and DR5 may be necessary but not sufficient to cause ABPA. Furthermore, Chauhan et al [4] demonstrated that Asp f l allergen has a low-affinity of binding to HLA-DR. This is consistent with Th2 T cell response previously reported by others in that strong antigen HLA-DR-Ag-TCR affinity binding favored a Th1 cellular response whereas low affinity binding favored a Th2 humoral response [21-25]. Thus, we propose that antigen presenting cells bearing HLA-DR2/DR5 and with increased sensitivity to IL-4 stimulation may play a critical role in skewing the Th2 responses to *A. fumigatus *in ABPA.

We previously reported that in ABPA patients there was significant up-regulation of CD23 expression of IL-4 stimulated peripheral blood mononuclear cells [7]. Other investigators have identified IL-4Rα single nucleotide polymorphisms (SNP) that result in a gain-of-function of IL-4 stimulation [8-16]. We examined one extracellular and four cytoplasmic SNPs. In these studies, we observed IL-4Rα SNPs in 95% of ABPA patients. The extracellular IL-4 binding SNP, ile75val, was the most frequently identified SNP and was frequently homozygous. Hershey's group reported that the combination of two variants, ile75val and gln576arg, together resulted in elevated IL-4 dependent CD23 expression and risk of atopy and asthma severity which was not observed when these SNPs were present alone [16]. However, we observed the combination of ile75val and a cytoplasmic IL-4Rα SNP in only 28% of ABPA patients.

Other genetic risk factors have been proposed for the development of atopy, asthma and ABPA. In particular, in the development of increased asthma severity, investigators have reported that another combination of the ile75val IL-4Rα SNP and arg110gln IL-13 SNP has also been associated with allergy and asthma [26,27]. We have recently begum studies to examine the IL-13 SNP. Saxena et al [28] reported that ABPA patients with polymorphisms (ala91pro, arg94arg) in the collagen region of pulmonary surfactant protein A2 (SP-A2) had more elevated total IgE levels and higher percentages of eosinophilia than those observed in patients who lacked the SNPs. They also found that 80% of patients carrying both alleles had ABPA (P = 0.0079, OR = 10.4), while only 50% and 60% of patients carrying each allele, individually, were ABPA subjects, suggesting an additive effect.

IL-10 may also play a significant role in the development of atopy and ABPA. In previous studies, we observed increased IL-10 and IL-5 synthesis of Asp f2, f3 and f4 stimulated peripheral blood lymphocytes in CF and asthmatic ABPA patients [7]. Polymorphisms of the IL-10 promoter have been associated with risk of asthma [29] and ABPA [30]. In particular, Brourad et al [30] reported the association of the -1082GG genotype of IL-10 promoter and the colonization with *A. fumigatus *and the development of ABPA in CF. The -10822G polymorphism has been associated with increased IL-10 synthesis; whereas the -1082A allele has lower IL-10 synthesis. These investigators reported that 23–25% of CF patients were -1082GG, 49–53% -1082AG, and 24–26% -1082AA. There was significantly increased risk to become colonized with *A. fumigatus *and to develop ABPA in the -1082GG genotype group, which developed in 7% of the patients. Thus, one may hypothesize that antigen presenting cells (dendritic cells) expressing HLA-DR2/DR5 with increased IL-10 synthesis and increased sensitivity to IL-4 stimulation due to IL-4Rα polymorphisms may be responsible for driving an allergic inflammatory response to *A. fumigatus *in ABPA.

The major limitation of this study is the small numbers of ABPA patients studied. However, the frequency of IL-4Rα polymorphisms is so prevalent, further studies will likely confirm this preliminary study.

## Competing interests

The author(s) declare that they have no competing interests.

## Authors' contributions

APK conceived of the study, participated in its design and coordination, and drafted the manuscript; BK participated in the molecular genetic studies; JDC was instrumental in providing patient samples; and MRW participated in the molecular genetic studies. All authors read and approved of the final manuscript.

## References

[B1] Greenberger PA, Adkinson NF Jr, Yunginer JW, Busse WW, Bochner BS, Holgate ST, Simons FER (2003). Allergic bronchopulmonary aspergillosis. Allergy: Principles and Practice.

[B2] Stevens DA, Moss R, Kurup VP, Knutsen AP, Greenberger P, Judson MA, Denning DW, Crameri R, Brody A, Light M, Skov M, Maish W, Mastella G, the Cystic Fibrosis Foundation Consensus Conference (2003). Allergic bronchopulmonary aspergillosis in cystic fibrosis: Cystic Fibrosis Foundation Consensus Conference. Clin Infect Dis.

[B3] Chauhan B, Santiago L, Hutcheson PS, Schwartz HJ, Spitznagel E, Castro M, Slavin RG, Bellone CJ (2000). Evidence for the involvement of two different MHC class II regions in susceptibility or protection in allergic bronchopulmonary aspergillosis. J Allergy Clin Immunol.

[B4] Chauhan B, Santiago L, Kirschmann DA, Hauptfeld V, Knutsen AP, Hutcheson PS, Woulfe SL, Slavin RG, Schwartz HJ, Bellone CJ (1997). The association of HLA-DR alleles and T cell activation with allergic bronchopulmonary aspergillosis. J Immunol.

[B5] Chauhan BA, Knutsen AP, Hutcheson PS, Slavin RG, Bellone CJ (1996). T cell subsets, epitope mapping, and HLA-restriction in patients with allergic bronchopulmonary aspergillosis. J Clin Invest.

[B6] Khan SP, McClellan JS, Knutsen AP (2000). Increased sensitivity to IL-4 in patients with allergic bronchopulmonary aspergillosis and atopy. Inter Arch Allergy Immunol.

[B7] Knutsen AP, Hutcheson PS, Albers GM, Consolino J, Smick J, Kurup VP (2004). Increased sensitivity to IL-4 in cystic fibrosis patients with allergic bronchopulmonary aspergillosis. Allergy.

[B8] Hershey GKK, Friedrich MF, Esswein LA, Thomas ML, Chatila TA (1997). Association of atopy with a gain-of-function mutation in the interleukin-4 receptor a chain. N Engl J Med.

[B9] Mitsuyasu H, Izuhara K, Mao XQ, Gao PS, Arinobu Y, Enomoto T, Kawai M, Sasaki S, Dake Y, Hamasaki N, Shirakawa T, Hopkin JM (1998). Ile50Val variant of IL4R alpha upregulates IgE synthesis and associates with atopic asthma. Nat Genet.

[B10] Deichmann K, Bardutsky J, Forster J, Heinzmann A, Kuehr J (1997). Common polymorphisms in the coding part of the IL-4 receptor gene. Biochim Biophys Acta.

[B11] Kruse S, Japha T, Tedner M, Sparholt SH, Forster J, Kuehr J, Deichmann KA (1999). The polymorphisms S503P and Q576R in the interleukin-4 receptor alpha gene are associated with atopy and influence the signal transduction. Immunology.

[B12] Ober C, Leavitt SA, Tsalenko A, Howard TD, Hoki DM, Daniel R, Newman DL, Wu X, Parry R, Lester LA, Solway J, Blumenthal M, King RA, Xu J, Meyers DA, Bleecker ER, Cox NJ (2000). Variation in the interleukin-4 receptor alpha gene confers susceptibility to asthma and atopy in ethnically diverse populations. Am J Hum Genet.

[B13] Wjst M, Kruse S, Illig T, Deichmann K (2002). Asthma and IL-4 receptor alpha gene variants. Eur J Immunogenetics.

[B14] Rosa-Rosa L, Zimmermann N, Bernstein JA, Rothenberg ME, Khurana Hershey GK (1999). The R576 IL-4 receptor alpha allele correlates with asthma severity. J Allergy Clin Immunol.

[B15] Mitsuyasu H, Yanagihara Y, Mao X, Gao P, Arinobu Y, Ihara K, Takabayashi A, Hara T, Enomoto T, Sasaki S, Kawai M, Hamasaki N, Shirakawa T, Hopkin JM, Izuhara K (1999). Cutting edge: Dominant effect of Ile50Val variant of the human IL-4 receptor α-chain in IgE synthesis. J Immunol.

[B16] Risma KA, Wang N, Andrews RP, Cunningham CM, Ericksen MB, Bernstein JA, Chakraborty R, Hershey GK (2002). V75R576 IL-4 receptor alpha is associated with allergic asthma and enhanced IL-4 receptor function. J Immunol.

[B17] Hackstein H, Kluter H, Fricke L, Hoyer J, Bein G (1999). The IL-4 receptor alpha-chain variant Q576R is strongly associated with decreased kidney allograft survival. Tissue Antigens.

[B18] Bux J, Stein EL, Bierling P, Fromont P, Clay M, Stroncel D, Santoso S (1997). Characterization of a new alloantigen (SH) on the human neutrophil Fc gamma receptor IIIb. Blood.

[B19] Knutsen AP, Warrier MR, Noyes B, Consolino J (2005). Allergic bronchopulmonary aspergillosis in a patient with cystic fibrosis: diagnostic criteria when the IgE level is <500 IU/ml. Ann Allergy Asthma Immunol.

[B20] Verhoef A, Higgins JA, Thorpe CJ, Marsh SG, Hayball JD, Lamb JR, O'Hehir RE (1993). Clonal analysis of the atopic immune response to the group 2 allergen of Dermatophagoides spp.: identification of HLA-DR and -DQ restricted T cell epitopes. Inter Immunol.

[B21] Lamb JR, Higgins JA, Hetzel C, Hayball JD, Lake RA, O'Hehir RE (1995). The effects of changes at peptide residues contacting MHC class II T-cell receptor on antigen recognition and human Th0 cell effector function. Immunol.

[B22] Tsitoura DC, Verhoef A, Gelder CM, O'Hehir RE, Lamb JR (1996). Altered T cell ligands derived from a major house dust mite allergen enhance IFN-γ but not IL-4 production by human CD4^+ ^T cells. J Immunol.

[B23] Pfeiffer C, Stein J, Southwood S, Ketelaar H, Sette A, Bottomly K (1995). Altered peptide ligands can control CD4 T lymphocyte differentiation in vivo. J Exp Med.

[B24] Evavold BD, Sloan-Lancaster J, Hsu BL, Allen PM (1993). Separation of T helper 1 clone cytolysis from proliferation and lymphokine production using analog peptides. J Immunol.

[B25] Racioppi L, Ronchese F, Matis LA, Germain RN (1993). Peptide-major histocompatibility complex class II complexes with agonist/antagonist properties provide evidence for ligand related differences in T cell receptor dependent intracellular signaling. J Exp Med.

[B26] Chen W, Ericksen MB, Hershey GKK (2004). Functional effect of the R110Q IL13 genetic variant alone and in combination with IL4RA genetic variants. J Allergy Clin Immuno.

[B27] Vladich FD, Brazille SM, Stern D, Peck ML, Ghittoni R, Vercelli D (2005). IL-13 R130Q, a common variant associated with allergy and asthma, enhances effector mechanisms essential for human allergic inflammation. J Clin Invest.

[B28] Saxena S, Madan T, Shah A, Muralidhar K, Sarma PA (2003). Association of polymorphisms in the collagen region of SP-A2 with increased levels of total IgE antibodies and eosinophilia in patients with allergic bronchopulmonary aspergillosis. J Allergy Clin Immunol.

[B29] Karjalainen J, Hulkkonen J, Nieminen MM, Huhtala H, Aromaa A, Klaukka T, Hurme M (2003). Interleukin-10 gene promoter region polymorphism is associated with eosinophil count and circulating immunoglobulin E in adult asthma. Clin Exp Allergy.

[B30] Brouard J, Knauer N, Boelle P-Y, Corvol H, Henrion-Caude A, Flamant C, Bremont F, Delaisi B, Duhamel J-F, Marguet C, Roussey M, Miesch M-C, Chadelat K, Boule M, Fauroux B, Ratjen F, Grasemann H, Clement A (2005). Influence of interleukin-10 on airways colonization by Aspergillus fumigatus in cystic fibrosis patients. J Infect Dis.

